# Efficacy of Cathelicidin LL-37 in an MRSA Wound Infection Mouse Model

**DOI:** 10.3390/antibiotics10101210

**Published:** 2021-10-05

**Authors:** Oriana Simonetti, Oscar Cirioni, Gaia Goteri, Guendalina Lucarini, Elżbieta Kamysz, Wojciech Kamysz, Fiorenza Orlando, Giulio Rizzetto, Elisa Molinelli, Gianluca Morroni, Roberto Ghiselli, Mauro Provinciali, Andrea Giacometti, Annamaria Offidani

**Affiliations:** 1 Clinic of Dermatology, Department of Clinical and Molecular Sciences, Polytechnic University of Marche, 60020 Ancona, Italy; molinelli.elisa@gmail.com (E.M.); a.offidani@staff.univpm.it (A.O.); 2 Clinic of Infectious Diseases, Department of Biomedical Sciences and Public Health, Polytechnic University of Marche, 60020 Ancona, Italy; o.cirioni@staff.univpm.it (O.C.); a.giacometti@staff.univpm.it (A.G.); 3 Division of Pathological Anatomy, Department of Biomedical Sciences and Public Health, Polytechnic University of Marche, 60020 Ancona, Italy; g.goteri@staff.univpm.it; 4 Department of Clinical and Molecular Sciences-Histology, Polytechnic University of Marche, 60020 Ancona, Italy; guendalina.lucarini@staff.univpm.it; 5 Team of Chemistry of Biological Macromolecules, Department of Molecular Biotechnology, Faculty of Chemistry, University of Gdańsk, 80-308 Gdańsk, Poland; elzbieta.kamysz@ug.edu.pl; 6 Department of Inorganic Chemistry, Faculty of Pharmacy, Medical University of Gdańsk, 80-416 Gdańsk, Poland; kamysz@gumed.edu.pl; 7 Experimental Animal Models for Aging Unit, Scientific Technological Area, IRCCS INRCA, 60100 Ancona, Italy; f.orlando@inrca.it (F.O.); m.provinciali@inrca.it (M.P.); 8 Unit of Microbiology, Department of Biomedical Sciences and Public Health, Polytechnic University of Marche, 60020 Ancona, Italy; g.morroni@staff.univpm.it; 9 Surgical Clinic, Department of Experimental and Clinical Medicine, Polytechnic University of Marche, 60020 Ancona, Italy; r.ghiselli@staff.univpm.it

**Keywords:** animal models, cathelicidin, LL-37, VEGF, wound

## Abstract

Background: LL-37 is the only human antimicrobial peptide that belongs to the cathelicidins. The aim of the study was to evaluate the efficacy of LL-37 in the management of MRSA-infected surgical wounds in mice. Methods: A wound on the back of adult male BALB/c mice was made and inoculated with *Staphylococcus aureus*. Two control groups were formed (uninfected and not treated, C0; infected and not treated, C1) and six contaminated groups were treated, respectively, with: teicoplanin, LL-37, given topically and /or systemically. Histological examination of VEGF expression and micro-vessel density, and bacterial cultures of wound tissues, were performed. Results: Histological examination of wounds in the group treated with topical and intraperitoneal LL-37 showed increased re-epithelialization, formation of the granulation tissue, collagen organization, and angiogenesis. Conclusions: Based on the mode of action, LL-37 has a potential future role in the management of infected wounds.

## 1. Introduction

*S. aureus* is one of the most frequent pathogens involved in skin and soft tissue infections (SSTIs), which includes hospital-acquired surgical site infections (SSIs) and community-acquired (CA) infections. Specifically, complicated SSTIs (cSSTI) affecting deep tissues, are the most severe infections, commonly observed in hospitalized patients [1]. Drug-resistant bacteria, such as methicillin-resistant *Staphylococcus aureus* (MRSA), are the most frequent aetiological agents in cSSTIs, and are associated with higher mortality and morbidity, as well as the need for intensive care unit (ICU) admission [2].

The glycopeptide family of antibiotics, to which teicoplanin also belongs, remains a mainstay option for suspected severe SSTI and invasive MRSA infections [3], even though there are multiple alternatives, and it has a potential for renal toxicity, Unfortunately, extensive antibiotic use in healthcare and agriculture in recent years [4] has determined a selection pressure on bacteria leading to a strong increase [5,6] in the emergence of MRSA isolates with reduced susceptibility to teicoplanin [7].

Therefore, the need to find new therapies with new mechanisms of action has stimulated medical research. In this regard, antimicrobial peptides (AMPs) represent a new therapeutic perspective, as they are derived from natural molecules of innate immunity, showing a broad-spectrum activity against pathogenic bacteria, yeast, viruses, and fungi, [8,9,10,11]. Many AMPs kill pathogens by disruption of membrane integrity and are, thus, thought to be less likely to induce resistance [12]. Furthermore, conventional antimicrobial molecules may benefit synergically from the association with AMPs, contributing to the reduction of resistance formation in bacteria and/or allow to restore sensitivity to conventional treatments [13,14,15,16,17,18]. Several families of AMPs have been found, among them the cathelicidins [19]. Approximately 30 types of cathelicidins have been identified in mammals, but hCAP-18 is the only component present in humans [20]. hCAP-18 (18 kDa), is processed, by specific serine proteases, to bioactive cathelicidin LL-37 [20] in many cells, such as epithelial cells, neutrophils, macrophages, natural killer cells, monocytes, dendritic cells, mast cells, and lymphocytes [21]. Its mechanism of action is based on the bond between its positive charges and the negative charges of the phospholipids in the bacterial membrane. Bacterial lysis occurs due to altered permeability of the bacterial membrane with transmembrane pores [22]. In fact, LL-37 showed a broad-spectrum against several different pathogens, such as Gram-positive and Gram-negative bacteria [23], fungi [24,25,26] and viruses [27]. Moreover, LL-37 revealed other biological activities, such as regulation of responses to inflammation, showing both pro-inflammatory and anti-inflammatory effects [28].

In addition, LL-37 demonstrated an important activity in wound closure [29,30] and angiogenesis [29,30,31].

Thus, LL-37 not only has wide antimicrobial activity, but is also a good candidate for the well-established efficacy in cutaneous healing in the treatment of infected cutaneous wounds.

To evaluate the efficacy of LL-37, we studied experimental mouse models with MRSA-infected surgical wounds, considering healing parameters such as collagen organization, degree of re-epithelialization, granulation tissue formation, and VEGF expression. Moreover, we compared our results with data from animals that received teicoplanin.

## 2. Results

In the present study, we investigated the efficacy of LL-37 in a mouse model of surgical wound infection, in comparison with teicoplanin-treatment mice. The main outcome measures in the study were quantitative cultures of excised tissues, the histological examination of injured areas, assessment of micro-vessel density, and VEGF expression by endothelial cells.

### 2.1. Cytotoxicity Assay

LL-37 did not affect cell proliferation. XTT of cells treated with the peptide was comparable with the control with no statistically significant differences.

### 2.2. Quantitative Cultures of Excised Tissues

**Figure 1:**
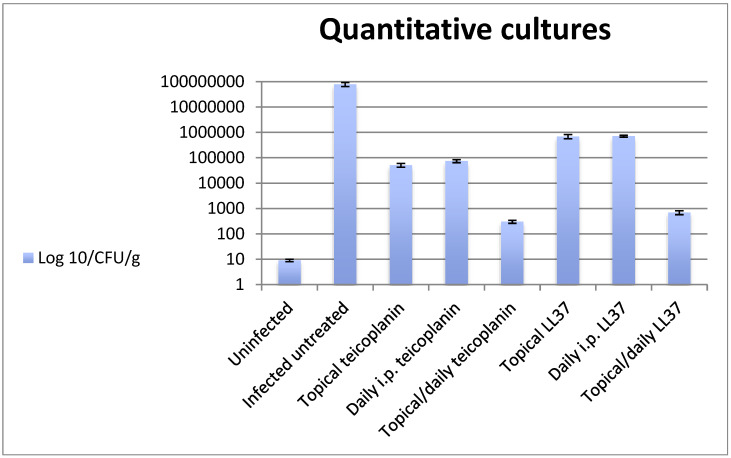
Bacterial growth in untreated and treated animals.

The quantitative cultures data are shown in Figure 1. Mean bacterial numbers in the challenged but untreated and infected control (7.8 × 10^7^ ± 1.4 × 10^7^ CFU/g) were significantly higher than those recovered from all the treatment groups. Topical teicoplanin and LL37 showed good antimicrobial efficacy (5.1 × 10^4^ ± 0.9 × 10^4^ CFU/g; 6.9 × 10^5^ ± 1.3 × 10^5^ CFU/g, respectively). When we considered i.p. treatments, the highest inhibition of bacterial load was obtained in the group that received i.p. teicoplanin (7.4 × 10^4^ ± 1.0 × 10^4^ CFU/g) (*p* < 0.01), while i.p. LL37 reduced bacterial numbers to 7.1 × 10^5^ ± 0.6 × 10^5^ CFU/g. When topical treatment was combined with i.p. treatment, the positive interaction produced bacterial counts of 3.0 × 10^2^ ± 0.4 × 10^2^ CFU/g for topical teicoplanin plus i.p. teicoplanin and 6.9 × 10^2^ ± 1.2 × 10^2^ CFU/g for topical LL37e and i.p. LL37, with statistically significant difference versus the control infected and not treated group (*p* < 0.001) and versus the singly treated groups (*p* < 0.05).

### 2.3. Evaluation of Excised Samples by Histology

**Figure 2:**
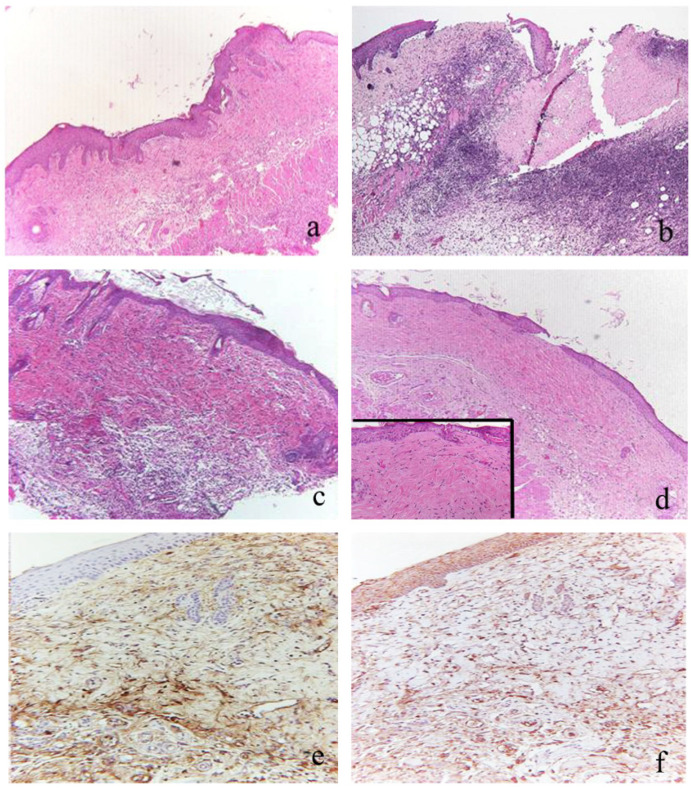
Histological sections of wounds from a not-infected control (**a**) and from an MRSA-infected and not-treated control (**b**). The uninfected control shows a complete re-epithelization and dermal collagenization, whereas the MRSA-infected and untreated wound is still not completely covered by epidermis without regular deposition of collagen fibers. Efficacy of different combined (topical and parenterally) treatments (in (**c**), teicoplanin, (**d**), LL37) on the MRSA-infected wounds: all treatment obtained an acceleration of the infected wound repair with a complete epidermal lining, a low number of inflammatory cells, and moderate dermal fibrosis. Treatments with LL37 (**d**) shows the highest degree of fibrosis in the dermis and subcutaneous tissue. Features of wound repair associated with LL37 (insert) at high power. Treatments (topically and parenterally) with LL37 show a significant fibrosis and increased angiogenesis in the dermis. CD31 (**e**) and VEGF (**f**) expression in MRSA-infected wound treated with LL37, topically and parenterally (**e**): several immunostained vessels and cells are evident. ((**a**–**d**) original magnification 100×, Insert original magnification 150×, H&E staining; (**e**,**f**) original magnification 200×, Immuno-Peroxidase).

Wound healing in the controls represented by uninfected animals showed a mature granulation tissue rich in all the typical cellular elements and a good organization of collagen fibers (Figure 2a). Conversely, the infected untreated mice displayed a significantly delayed wound healing process due to an immature granulation tissue with a few fibroblasts and a poor collagen fiber arrangement (Figure 2b). The evaluation of wound repair according to the wound healing score is illustrated in Table 1; Table 2. After administration of the treatments, infected wounds showed a positive healing course (Table 1, Figure 2c,d). In particular, the wounds treated with topical and parenterally LL37 (Figure 2d) were characterized by a sensible decrease of fibrinous exudation and the presence of a mature collagen organization (Figure 2d insert), if compared to wounds treated with teicoplanin (Table 1). Moreover, they showed a good angiogenesis process with a significant increase of micro-vessel density (Figure 2e) and VEGF expression (Figure 2f) compared to the teicoplanin-treated wounds (Table 2).

## 3. Discussion

The incidence of MRSA continues to increase with the constant emergence of new strains [32]. Moreover, it has been found that the inflammatory response after bacterial infection is a contributing factor to the clinical burden of *S. aureus* skin infections, rather than the bacterial load. [33].

The skin is the widest organ in the human body and is frequently affected by wounds, commonly caused by burns, trauma, and skin diseases. [34] In particular, physical and biochemical defensive barriers arise from the association of keratinocytes (KC) with sweat products, lipids, and antimicrobial peptides (AMPs). The latter include cathelicidins, which have been proven to be activated by infections, lesions or inflammation of the skin [35].

The aim of this study was to evaluate the role of synthetic LL37 in the treatment of skin and surgical wound infections in a mouse model induced with a strain of MRSA.

Regarding LL-37 antimicrobial activity, our results highlighted the potential efficacy of synthetic LL-37 treatment. Based on CFU counts of excised tissues, we have observed that topical LL-37 treatment was already effective in reducing bacterial counts; although, the best outcome was achieved by combining systemic and topical LL37, with results comparable to those observed in the topical and systemic teicoplanin treatment group. Our results exhibited that the administration of LL-37 intraperitoneally to the infected mice are safe with no toxic side effect, opening a new horizon in the treatment of antibiotic-resistant strains [36]. AMPs have great potential as bactericidal molecules, but their antimicrobial and wound healing effects could be limited by AMP susceptibility to degradation after topical application [37]. A previous report on a postoperative MRSA infection of a femoral fracture in a rabbit model showed that two days after LL-37 injection, biofilm colony counts showed significant changes compared to the Cefalexin groups [38]. In addition, another study showed that LL-37 could eradicate S. aureus both extra- and intracellularly more rapidly than conventional antibiotics [37].

It was reported that the antibacterial properties of LL-37 can be reduced by certain biological fluids containing serum and glycosaminoglycans [38]; however, the removal of N-terminal hydrophobic amino acids from LL-37 appears to reduce the inhibitory effect of the serum without impairing its antimicrobial properties [39].

In a previous study on cathelicidins against different animal species, LA–MRSA showed that antimicrobial resistance genes of common staphylococci do not reduce the antimicrobial activity of these peptides [40]. The mechanism of action of LL-37 begins, like other AMPs, with an electrostatic interaction between the anionic components of the bacterial membrane and cationic peptides, followed by inclusion in the bacterial lipid membrane, resulting in transmembrane toroidal pore-forming and membrane disruption [41].

Although it was reported that AMPs can develop resistance mechanisms in MRSA [42], the effect on the minimum inhibitory concentration of AMPs (2–30 times increase) is less dramatic than for antibiotics (100–1000 times increase) [41,43], this is because their main targets are the intracellular molecules and cytoplasmic membrane [44,45,46]. Moreover, AMP resistance acquired by surface molecule change [47] or proteolytic cleavage [48] is confined and compared to the conventional drugs, needs longer times.

Additionally, our results indicated that LL-37 showed a stronger effect than teicoplanin on the wound healing process in MRSA-infected mice. We demonstrated that LL-37, after topical and parenteral administration, enhanced the wound closure via stimulation of granulation tissue formation associated with a better organized collagen deposition and reconstitution of the epithelium, in comparison with the teicoplanin treatment group. These findings underlined that LL-37, besides its antimicrobial properties, is effective in promoting wound repair.

Wound healing is a complex process that restores the integrity of the skin, forming new connective tissue from granulation tissue and new epithelial tissue to close the wound. One of the main factors contributing to wound healing is the migration of keratinocytes in the wound bed. LL-37 induces a migratory phenotype in keratinocytes [30,49] and this effect is mediated by the transactivation of EGFR [49]. On the other hand, the EGFR pathway represents an important step and a positive indicator in wound healing [50,51].

The formation of granulation tissue begins a few hours after skin injury, orchestrated first by neutrophils migration and later monocyte recruitment and their differentiation to macrophages, leading to a release of chemoattractants and growth factors such as vascular endothelial growth factor (VEGF) [52,53].

VEGF allows the maintenance of physiological vascular homeostasis in different tissues by creating nutritional support through increased vascular permeability of plasma proteins [54,55]. It also plays an important role not only in tumor growth and metastasis, but also in wound healing [56,57,58]. Our results clearly support the hypothesis that human LL-37 can promote important angiogenesis. In previous studies LL-37, as well as its antimicrobial activities, showed its influence on both innate and adaptive immunity [59]. Therefore, LL-37, activating neutrophils and monocytes, promotes the release of angiogenic mediators and stimulates new blood vessel production [60]. It was suggested that the neovascularization, associated with LL-37, could be due to a direct effect on endothelial cells, through the involvement of the specific G-protein-coupled formyl peptide receptor-like 1 (FPRL-1) [59]. FPRL-1 is present in macrophages, neutrophils, lymphocytes (primarily NK cells, B cells, and γδT lymphocytes), keratinocytes, as well as in epithelial and endothelial cells [30].

## 4. Materials and Methods

### 4.1. Reagents

Fmoc-amino acids and Fmoc-Ser(tBu)-PEG-PS resin (0.17 meq/g) were purchased from Applied Biosystems (Foster City, CA, USA), and ChemImpex (Wood Dale, IL, USA). All other reagents and solvents were of synthesis grade.

### 4.2. Bacterial Strains and Drugs

*S. aureus* ATCC 43300, the reference MRSA strain, was used in the mouse infection model.

Teicoplanin (Aventis Pharma S.p.A., Rome, Italy) was diluted in accordance with the manufacturer’s recommendations, yielding 10 mg/mL of stock solution.

The peptide LL-37 (LLGDFFRKSKEKIGKEFKRIVQRIKDFLRNLVPRTES) was synthesized manually by the solid-phase method using 9-fluorenylmethoxycarbonyl (Fmoc) chemistry on a 2-chlorotrityl chloride resin (1.0–1.6 mmol Cl^−^ g^−1^ resin, 100–200 mesh, Iris Biotech GMBH, Wiesbaden, Germany) according to procedures described in our previous works [60,61]. The purity of LL-37 after purification was at least 98%, as determined by analytical reversed-phase high-performance liquid chromatography (RP-HPLC). Its identity was confirmed by the electrospray ionization mass spectrometry (ESI-MS).

### 4.3. Ethics

In vivo experiments were approved by the Institutional Animal Care Committee of the Ministry of Health and by the Animal Research Ethics Committee of IRCCS-INRCA (Istituto di Ricovero e Cura a Carattere Scientifico—Istituto nazionale di Riposo e Cura per Anziani) 767/2016 Pr 28/07/2016.

### 4.4. Animals

In our study, we utilized six-month-old BALB/c mice weighing from 28–30 g from the Specific Pathogen Free (SPF) animal facility of INRCA (Italian National Centre on Health and Science on Aging, Ancona, Italy) and the procedures were conducted in conformity with the national (Legislative Decree n. 26, 4 March 2014; Authorization n. 767/2016-PR issued 28 July 2016, by the Italian MoH) and international law and policies (EEC Council Directive 2010/63/EU) as previously described (51).

Eighty animals were included in the research, divided into eight groups (each composed of ten mice): an uninfected group (C0, sham control); an infected but not treated group (C1); a teicoplanin group (infected and topically treated, C2); an intraperitoneal teicoplanin group (infected and treated daily with teicoplanin, C3); a topical teicoplanin and daily intraperitoneal teicoplanin (infected and treated topically and systemically with teicoplanin, C4) a topical LL-37 group (infected and topically treated C5); an intraperitoneal LL-37 group (infected and treated daily with LL37, C6), a topical LL-37 and intraperitoneal LL-37 (infected and treated topically and systemically with LL37, C7).

The MRSA ATCC43300 were grown in brain hearth infusion and diluted in saline to a final concentration of 5 × 10^7^ CFU/mL, prepared freshly at the time of intervention. Mice were anesthetized by an intramuscular injection of ketamine (50 mg/kg of body weight) and xylazine (8 mg/kg of body weight), the hair on their back was shaved, and the skin was cleansed with 10% povidone-iodine solution (no animals dropped out due to infection or anesthetics). One full-thickness wound was established through the panniculus carnosus on the back subcutaneous tissue of each animal. A small piece of gauze was placed over each wound and then inoculated with 200 μL of previously diluted bacterial culture; in the control group, the gauze was soaked only with sterile saline solution. The pocket was closed by means of skin clips. This procedure resulted in a local abscess at 24 h. One wound was created per animal. The animals were returned to individual cages and thoroughly examined daily. After 24 h, the wound was opened and washed with saline, the gauze was removed, and treatment started [51]. The group were randomized to receive C2) topical teicoplanin (7 mg/Kg), topical LL-37 (1 mg/Kg), C5); intraperitoneal LL-37 (1 mg/Kg; C6), daily intraperitoneal teicoplanin (7 mg/Kg; C4); topical LL-37 and intraperitoneal LL-37 at the same dosage of 1 mg/Kg (C7). Drug dosages were determined according to our previous studies and pharmacokinetic and pharmacodynamic information from other experimental studies [62,63]

We made comparisons with two control groups (uninfected and not treated, C0; infected and not treated (C1).

After 14 days, animals were euthanized and a 1 × 2 cm area of skin, including the wound, was excised aseptically for histological and Western blot examination (see below) and for bacterial count. For the bacterial analysis, the samples were weighted and then homogenized in 1 mL of phosphate-buffered saline (PBS) using a stomacher. Quantitation of viable bacteria was performed by culturing serial dilutions of the bacterial suspension on mannitol-salt agar plates at 37 °C for 24–48 h. The limit of detection for this method was approximately 10 CFU/mL.

### 4.5. Cytotoxicity Assay

For cytotoxicity assay, HaCaT cells were seeded in a 96-well plate. The cytotoxicity of LL37 was determined by XTT cell proliferation assay, using 10 mg/L of LL-37, after 24, 36, and 48 h. Cells cultured without drugs were used as the control.

#### 4.5.1. Histology

The healing process of infected and uninfected wounds was evaluated by histological analysis. The excised samples were routinely processed [10]. We observed the histological sections under light microscopy, and we assessed the degree of wound healing by considering the epithelial re-epithelialization, granulation tissue formation, inflammation, angiogenesis, and collagen organization as described in previous studies [50,64] (Table 3).

#### 4.5.2. Angiogenesis Evaluation

Angiogenesis was evaluated on paraffin-embedded tissue sections by immunohistochemistry, assessing the micro-vessel density (MVD) and the endothelial cell VEGF expression according to Simonetti et al. [50]. The sections were incubated overnight at 4 °C with the anti-VEGF-C-1 (diluted 1:200, Santa Cruz, CA, USA) and anti-CD31/PECAM-1 (diluted 1:20, Santa Cruz, CA, USA) antibodies. The number of CD31-positive small vessels (MVD) was counted under a light microscope at a magnification of 400× covering an area of 0.16 mm^2^ per field. We considered positive any brown stained endothelial cell or endothelial cell cluster clearly separated from adjacent micro-vessels. MVD was represented as number of counted micro-vessels per mm^2^ while positive VEGF cells were expressed as percentage over total cells counted in the selected fields. All counts were performed by one investigator three times for each sample and expressed as the obtained mean values.

### 4.6. Statistical Analysis

All results are presented as mean ± SD. Student’s *t*-test and analysis of variance (ANOVA) were performed for statistical analysis. Significance was accepted when the *p* value was <0.001.

## 5. Conclusions

In conclusion, in our mouse model, we evidenced that LL37, through its interaction with bacteria membrane lipids, shows good antimicrobial activity, suggesting its role as an encouraging option for new treatments in MRSA infections. Moreover, our results clearly support the important role of human LL-37 in promoting angiogenesis and the healing process.

## Figures and Tables

**Table 1:** Summary of the biological impact of different treatment modalities on wound healing parameters at day 7 post-wounding. Data are expressed as the mean score ± SD.

Treatment	Re-Epithelialization	Granulation Tissue	Collagen Organization
C0 Uninfected and no treatment, control group	2.67 ± 0.44	2.93 ± 0.87	2.57 ± 0.38
C1 Infected and no treatment, control group	1.11 ± 0.51	2.04 ± 0.62	0.72 ± 0.35
C2 Infected and treated with topical teicoplanin	2.48 ± 0.43	2.41 ± 0.69	2.09 ± 0.54
C3 Infected and treated with daily i.p. teicoplanin	2.00 ± 0.49	2.19 ± 0.71	1.91 ± 0.66
C4 Infected and treated with topical teicoplanin and daily i.p. teicoplanin	2.63 ± 0.67	2.85 ± 0.48	2.32 ± 0.60
C5 Infected and treated with topical LL37	2.54 ± 0.52 *	2.81 ± 0.74 *	2.49 ± 0.49 *
C6 Infected and treated with daily i.p. LL37	2.38 ± 0.63 *	2.42 ± 0.49 *	2.16 ± 0.33 *
C7 Infected and treated with topical LL37 and daily i.p. LL37	2.70 ± 0.52 *	2.90 ± 0.74 *	2.73 ± 0.49 *

Anova test, * *p* < 0.001 vs. teicoplanin-treated groups and untreated infected group.

**Table 2:** Micro-vessels density (MVD) and number of VEGF positive cells at day 7 post-wounding. Data are expressed as the mean score ± SD.

Treatment	MVD Expression (Small Vessels/mm^2^)	VEGF Expression (Positive Cells/mm^2^)
C0 Uninfected and no treatment, control group	230.53 ± 30.21	467.52 ± 38.76
C1 Infected and no treatment, control group	155.36 ± 33.68	294.27 ± 46.22
C2 Infected and treated with topical teicoplanin	204.66 ± 49.76	359.86 ± 135.20
C3 Infected and treated with daily i.p. teicoplanin	215.03 ± 66.48	373.78 ± 122.37
C4 Infected and treated with topical teicoplanin and daily i.p. teicoplanin	226.59 ± 57.33	456.38 ± 45.78
C5 Infected and treated with topical LL37	265.63 ± 31.6 *	470.82 ± 46.61 *
C6 Infected and treated with daily i.p. LL37	227.52 ± 38.9 *	459.24 ± 37.6 *
C7 Infected and treated with topical LL37 and daily i.p. LL37	271.77 ± 54.9 *	493.47 ± 68.18 *

Anova test, * *p* < 0.001 vs. teicoplanin-treated groups and untreated infected group.

**Table 3:** Score of morphological features.

Score	Re-Epithelialization	Granulation Tissue Formation	Collagen Organization
0	None	None	None
1	Migrating epithelial cells	Hypo cellular with few vessels	Trace
2	Partial stratum corneum	Many vessels and some cells	Slight
3	Hypertrophic stratum corneum	Many fibroblasts, some fibers	Moderate
4	Complete and normal stratum corneum	More fibers, few cells	Marked

## Data Availability

Not applicable.
